# Interferons

**DOI:** 10.1155/S1064744995000561

**Published:** 1995

**Authors:** Lawrence J. Eron

**Affiliations:** Infectious Diseases/Internal Medicine Kauai Medical Group, Inc. 3-3420 Kuhio Highway Suite B Liue HI 96766-1098 USA


Infectious Diseases in Obstetrics and Gynecology 3:176–178 (1995)
© 1996 Wiley-Liss, Inc.

### Interferons

**Lawrence J. Eron**
*Infectious Diseases/Internal Medicine, Kauai Medical Group, Inc., Lihue, HI*

KEY WORDS
Human papillomavirus, condyloma acuminatum, herpesvirus, human immunodeficiency virus, antivirals

Interferons, naturally occuring glycoproteins with molecular weights of approximately 20,000 daltons, possess antiviral, antiproliferative, and immune-stimulating properties. They are classified into 3 antigenically distinct forms that are derived from different cell types. Alpha (α) interferons are derived from leukocytes, beta (β) interferons from fibroblasts, and gamma (γ) interferons from T lymphocytes. They have been approved for use in the treatment of condylomata acuminata, hepatitis B and C, and hairy-cell leukemia.

While naturally occurring α interferons can be obtained from pooled leukocytes derived from whole blood and then stimulated to produce interferon [α-n3 = Alferon™ (Purdue Frederick Company, Norwalk, CT); α-N1 = Wellferon™ (Pacific Pharmacy, San Francisco, CA)], they can also be produced through recombinant DNA technology [α-2a = Roferon™ (Roche Laboratories, Nutley, NJ); α-2b = Intron A™ (Schering Corporation, Kenilworth, NJ)]. α-2a and α-2b differ by only 1 amino acid in position −23. The natural interferons are really mixtures of 14 or more types, while the recombinant types are pure quantities of a single species of interferon.

### MECHANISM OF ACTION

As indicated above, interferons are antiviral, antiproliferative, and immune-stimulating. The mechanism by which interferons block viral replication is by inducing more than 24 different cellular proteins that degrade viral RNA and interfere with viral protein synthesis. The antiproliferative activity of interferons has been utilized in slowing the growth of tumor cells. This cytostatic effect is achieved by a modulation of the levels of cellular 2′,5′-oligoadenylate synthetase and of cell oncogenes such as c-myc, c-fos, and c-H-ras. The immune-stimulating properties contribute to the antiviral and antitumor activities by enhancing the expression of cell surface antigens, such as major histocompatibility complex antigens. Interferons contribute to the lysis of virus-infected and tumor cells by activating macrophages, natural killer cells, and cytotoxic T lymphocytes, as well as by cytokine induction.[1]

### PHARMACOKINETICS

The intramuscular administration of interferon produces peak plasma concentrations in 4–8 h. The elimination half-life is between 2 and 3 h.[2] However, the physiologic half-life may extend far beyond this time. Little is known of the pharmacokinetics of the intralesional (intradermal) or topical administration. The activity is expressed in international units (I.U.) and vials containing 3, 5, 10, 25, and 50 million I.U. of recombinant interferon are commercially available. Once reconstituted with bacteriostatic water for injection, it is stable for 1 month if refrigerated. Natural interferons (Alferon™) are available in 5 million I.U. vials which are stable for 6 months if kept refrigerated. An 0.1 ml quantity (usually 1 million units) is injected intradermally with a number 30 needle into the base of each lesion 2–3 times weekly. An intradermal injection is performed in a similar manner to a purified protein derivative (PPD) injection. For intramuscular administration, usually 3 million I.U. is

Address correspondence/reprint requests to Dr. Lawrence J. Eron, Infectious Diseases/Internal Medicine, Kauai Medical Group, Inc., 3-3420 Kuhio Highway, Suite B, Lihue, HI 96766-1098.

Received November 8, 1995
Accepted November 10, 1995

injected either "regionally" into the anterior thigh or "systemically" into the deltoid muscle.

### SIDE EFFECTS

The most commonly reported side effects are flu-like symptoms including myalgias, headache, fever, chills, nausea, and fatigue. Transient leukopenia, occasional thrombocytopenia, and clinically insignificant abnormalities of liver function tests all readily reverse with discontinuation of therapy. The development of antibodies to interferon in 1% of the recipients appears to have no clinical significance. The flu-like side effects are reduced by limiting the total dose to 3 or, at most, 5 million I.U. per day and prophylactically administering acetaminophen immediately prior to therapy and again 6 h later when the chills and fever tend to occur. Another helpful technique is to administer the interferon late in the day, about 5 P.M. Since the chills and fever occur 6 h later, the patient can go to bed as usual and awaken in the morning free of side effects. With the subsequent administration of interferon, tachyphylaxis occurs with a lessening or disappearance of symptoms (except fatigue) by the second week. Interferon is contraindicated in pregnancy.

### SPECTRUM OF ANTIVIRAL ACTIVITY

Interferons, although they have no direct antiviral activity in vitro, stimulate cells of the immune system to develop antiviral activity and to produce antiviral substances. Interferons have a broad spectrum of antiviral activity with proven in vivo activity against human papillomavirus (HPV), hepatitis B and C viruses, the herpes virus group (herpes simplex, herpes zoster, cytomegalovirus), influenza virus, and human immunodeficiency virus. Within the HPV group, clinically equipotent activity has been demonstrated against the common anogenital HPV types 6, 11, 16, and 18.

### CLINICAL APPLICATIONS

Intralesionally injected interferon has demonstrated clinical efficacy in the treatment of condylomata acuminata in randomized, placebo-controlled, double-blind clinical trials.[3,4] However, its clinical usefulness is limited by several factors. First, while it cures up to 70% of the warts that are refractory to conventional therapy (laser, electrodesiccation, cryotherapy, curettage, trichloroacetic acid, podophyllotoxin, and 5-fluorouracil), approximately 25% of the "cured" warts recur,[3,4] undoubtedly due to the persistence of HPV in the adjacent normal tissue.[5] Second, the intralesional injection twice or thrice weekly is a cumbersome, labor-intensive method of administration that is not adaptable to cervical or vaginal disease. Two alternative approaches circumvent these limitations: 1) topical application of interferon or an interferon inducer and 2) regional or systemic administration of interferon.

After an early successful trial, topical interferon administration has not proved effective,[6] although recent work using the topically applied imiquimod, a heterocyclic amine that induces interferon production in peripheral blood mononuclear cells, has demonstrated efficacy in a placebo-controlled, double-blind trial.[7] The potential usefulness of a topical application in the treatment of cervical or vaginal disease and its lack of any systemic side effects make this compound an interesting one suitable for further clinical trials.

Regional (in the anterior thigh) or systemic (in the deltoid muscle) administration of interferon has been demonstrated to be effective in the treatment of hepatitis B and C (50% and 25% cure rates, respectively). However, it has not shown as much efficacy in the treatment of anogenital HPV infections. The cure rates of the systemic administration of interferons to treat condylomata acuminata have been disappointingly low.[8] Adjuvant therapy with systemic interferon did not increase the efficacy of cryotherapy in eradicating condylomata acuminata either during or following treatment.[9,10] However, in 2 randomized trials, when interferon was administered regionally in the anterior thigh, it increased the efficacy of laser therapy in treating vulvar condylomata acuminata.[11,12] Why a discrepancy exists between laser therapy and cryotherapy when interferon is used as an adjuvant chemotherapy is unclear. It may have to do with the ability of the laser to eradicate the viral reservoir in the adjacent normal tissue. Alternatively, it may reflect the possible efficacy of the regional route (in the anterior thigh) compared with systemic administration (in the deltoid muscle) of interferon.

Systemic interferon therapy did not eliminate HPV DNA or reduce the amount of DNA present when genital warts were treated systemically with $\alpha$ interferon.[13,14] Similarily, $\alpha$ interferon failed to eliminate HPV DNA from laryngeal papillomas that were treated systemically with interferon.[15] Furthermore, neither β interferon nor α interferon has shown greater efficacy when systemically administered as an adjuvant to cryotherapy.[16] Additionally, these interferons are less effective in the treatment of HIV-positive individuals, who have an especially high relapse rate.[17] These results suggest that systemically administered interferons work mainly as antiproliferative agents and have not yet fulfilled their antiviral and immune-stimulating potentials in the case of HPV infection.

### COST

A 3 million I.U. vial, enough for a single treatment, costs $31.50 (wholesale price). However, this cost does not include the cost of administering the interferon. It has been calculated to cost approximately $1,500 for a course of therapy including the cost of administration.[6,9] Purchasing interferon in larger quantities such as 5 million ($52.50), 10 million ($105), or 18 million ($189) I.U. vials does not reduce the cost.

### SUMMARY

Interferons are useful in treating recalcitrant or recurrent warts, but are certainly not first-line therapy. The intralesional administration is effective but limited to vulvar and penile disease. Regional administration in the anterior thigh may be useful as an adjuvant to superficial laser vulvectomy. Interferons have not been shown to have a systemic effect on anogenital warts when used either as primary therapy or as an adjuvant to cryotherapy. They have not been shown to reduce HPV DNA following systemic therapy. In early trials, a topical interferon inducer, imiquimod, appeared to be effective without systemic side effects. This form may also be useful in the treatment of vaginal and cervical disease.

### REFERENCE

1. Tyring S: Interferons: Biochemistry and mechanisms of action. Am J Obstet Gynecol 172:1350–1353, 1995.
2. Browder JF, Aranjo OE, Myer NA, Flowers FP: The interferons and their use in condylomata acuminata. Ann Pharmacother 26:42–45, 1992.
3. Eron LJ, Judson F, Tucker S, et al.: Interferon therapy for condylomata acuminata. N Engl J Med 315:1059–1064, 1986.
4. Friedman-Kien AE, Eron LJ, Conant M, et al.: Natural interferon alpha for treatment of condylomata acuminata. JAMA 259:533–538, 1988.
5. Ferenczy A, Mitao M, Nagai N, Silverstein SJ, Crum CP: Latent papillomavirus and recurring genital warts. N Engl J Med 313:784–788, 1985.
6. Kraus SJ, Stone KM: Management of genital infection caused by human papillomavirus. Rev Infect Dis 12:S620–632, 1990.
7. Eron LJ, Edwards L, Ferenczy A, Baker D, Fox T, Gayoso K: Treatment of genital warts with imiquimod cream (abstract no. 380 presented at the Infectious Diseases Society of America 1995 Annual Meeting). Clin Infect Dis 21:783, 1995.
8. Stone KM: Human papillomavirus infection and genital warts: Update on epidemiology and treatment. Clin Infect Dis 20:S91–97, 1995.
9. Handley JM, Horner T, Maw RD, Lawther H, Dinsmore WW: Subcutaneous interferon alpha 2a combined with cryotherapy vs. cryotherapy alone in the treatment of primary anogenital warts: A randomized observer blind placebo controlled study. Genitourin Med 67:297–302, 1991.
10. Eron LJ, Alder MB, O'Rourke JM, Rittweger K, DePamphilis J, Pizzutti DJ: Recurrence of condylomata acuminata following cryotherapy is not prevented by systemically administered interferon. Genitourin Med 69:91–93, 1993.
11. Reid R, Greenberg MD, Pizzuti DJ, Omoto KH, Rutledge LH, Soo W: Superficial laser vulvectomy. V. Surgical debulking is enhanced by adjuvant systemic interferon. Am J Obstet Gynecol 166:815–820, 1992.
12. Petersen CS, Bjerring P, Larsen J, et al.: Systemic interferon alpha-2b increases the cure rate in laser treated patients with multiple persistent genital warts: A placebo-controlled study. Genitourin Med 67:99–102, 1991.
13. Griffiths M, Sanderson D, Penn LK: Cervical epithelial abnormalities among women with vulval warts: No more common than among controls. Int J Gynaecol Cancer 2:49–51, 1992.
14. Reichman RC, Oakes D, Bonnez W, et al.: Treatment of condyloma acuminatum with three different interferon-α preparations administered parenterally: A double-blind, placebo-controlled trial. J Infect Dis 162: 1270–1276, 1990.
15. Steinberg B, Gallagher T, Stoler M, et al.: Persistence and expression of human papillomavirus during interferon therapy. Arch Otolaryngol Head Neck Surg 114:27–32, 1988.
16. Bonnez W, Oakes D, Bailey-Farchione A, et al.: A randomized, double-blind, placebo-controlled trial of systemically administered interferon-α, -β, or -γ in combination with cryotherapy for the treatment of condyloma acuminatum. J Infect Dis 171:1081–1089, 1995.
17. Douglas JM, Eron LH, Judson FN, et al.: A randomized trial of combination therapy with intralesional interferon-$\alpha_{2b}$ and podophyllin versus podophyllin alone for the therapy of anogenital warts. J Infect Dis 162:52–59, 1990.
